# Umbrella review protocol: Global burden and risk factors of erectile dysfunction in diabetic population

**DOI:** 10.1002/hsr2.2159

**Published:** 2024-05-30

**Authors:** Tegene Atamenta Kitaw, Biruk Beletew Abate, Befkad Derese Tilahun, Gizachew Yilak, Ribka Nigatu Haile

**Affiliations:** ^1^ Department of Nursing, College of Health Science Woldia University Woldia Ethiopia

**Keywords:** associated risk factors, diabetic patients, erectile dysfunction, global burden, protocol

## Abstract

**Background:**

Erectile dysfunction (ED) is no longer a whisper in the shadows; it's a rising tide threatening the sexual health of millions of men in different regions. This worrying trend shows no signs of slowing down, with projections claiming a staggering 322 million men globally could be affected in the near future. In the cases of diabetes, the condition worsens and has a potent cocktail of physical and psychological distress, chipping away at men's confidence, self‐esteem, and mental health. This urgent issue demands immediate attention and action. Thus, this umbrella review intended to estimate the current burden of ED and associated risk factors among diabetic patients in the global context.

**Methods:**

Following PRISMA guidelines will be searched for relevant Systematic Review and Meta‐analysis studies in PubMed, Embase, Scopus, Web of Science, Cochrane Database of Systematic Reviews, and Google Scholar. The quality of the included studies will be assessed using the new regress tool, the Assessment of Multiple Systematic Reviews 2 tool. To estimate the pooled prevalence of ED, we will employ a weighted inverse variance random‐effects model. We will further conduct subgroup analyses, assess heterogeneity and publication bias, and perform sensitivity analyses to strengthen the robustness of our findings. Prediction intervals will also calculated to estimate the range within which future observations will likely fall. In all statistical analyses, the statistical significance will be declared at *p* value < 0.05.

**Discussion:**

This umbrella review of systemic review and meta‐analysis will be the first to systematically explore and integrate evidence regarding the burden of ED and associated risk factors in the diabetic population in a global context. By estimating the worldwide burden and identifying risk factors of ED in this population, the study will contribute to uncovering the hidden burden. Thereby, the issue will get international attention to reduce its consequences on the sexual health of the diabetic population. Besides, it will also provide input and direction for future research outlook.

## 1 INTRODUCTION

The number of men who have erectile dysfunction (ED) is on the rise worldwide, posing a growing threat to their overall well‐being. This concerning trend is expected to continue, with projections indicating a staggering 322 million men globally,^1^ highlighting the urgency of addressing this issue. ED is referred to as a consistent inability to obtain or maintain an erection that is sufficient for sexual satisfaction.^2^

Several chronic conditions, including diabetes mellitus (DM), cardiovascular disease, and depression, exhibit a strong correlation with increased rates of ED, with DM playing a particularly significant role.^3^ The frequency of self‐reported ED among men without accompanying health conditions surged from 10% to 79% between the ages of 40 and 80. With the presence of diabetes, the likelihood of experiencing ED escalated significantly.^4^, ^5^, ^6^ Diabetes combined with an increase in age increases the risk of ED. For instance, while 50‐ and 75‐year‐old healthy men faced a 20% and 68% probability of ED respectively, those with conditions such as diabetes encountered a higher risk, reaching 41% and 85% respectively.^7^

In diabetes, ED stems from multiple mechanisms. Chronically high blood sugar levels lead to damage in blood vessels (endothelial dysfunction),^8^ buildup of harmful sugar byproducts (advanced glycation end products), increased cell waste and damage (oxidative stress), and malfunctioning nerves (neuropathy). These factors disrupt the normal erectile response, making it difficult to achieve and maintain an erection.^9^ Diabetes can cause two types of nerve damage, peripheral and autonomic, that both contribute to ED. Peripheral neuropathy messes up signals between the penis and the brain, making it harder for the body to get excited.^10^ It also weakens the muscles that control blood flow in the penis, making it difficult to get and keep an erection.^11^

ED is more than just a physical issue; it can significantly impact a man's mental and emotional well‐being, affecting his confidence, relationships, and overall happiness.^12^ ED in people with diabetes may signal silent heart disease and predict future cardiovascular events while also impacting mental well‐being by lowering self‐esteem and increasing anxiety and depression.^13^ ED, while not directly life‐threatening, can cause significant distress and impact quality of life. Along with the stigma surrounding the problem, it often leads to underreporting. Thus, early detection and management of factors contributing to ED remain challenging.^14^

By 2025, an estimated 322 million men will be diagnosed with ED, further emphasizing the critical nature of this growing public health issue.^15^ The prevalence of ED in diabetic patients ranges from 35% to 90%.^16^, ^17^ Patients with ED often face a cascade of negative impacts, including a diminished quality of life,^18^, ^19^ anxiety as their sexual abilities decline, strained partner relationships due to mistrust, general unhappiness, and even fear of losing their partner's support.^20^ The economic burden of ED should not be overlooked either. Employers face higher absenteeism and lower productivity among employees struggling with ED. An observational study found men with ED missing work twice as often and experiencing greater work impairment compared with colleagues without ED.^21^ However, taking control of the underlying condition can help alleviate some of these challenges.

ED is a common complication of diabetes, affecting millions of men worldwide. Yet, despite its significant impact on diabetic patients, it remains largely a hidden burden, shrouded in stigma and shame. This lack of awareness and open discussion has far‐reaching consequences, hindering early detection, treatment, and, ultimately, the prevention of long‐term complications. Thus, understanding ED among diabetic patients in a global context can have contribute to improving the health and well‐being of diabetic patients worldwide. It is also essential to gain global attention, promoting global collaboration to prevent the silent burden. Thus, this umbrella review aims to estimate the burden of ED and associated risk factors in diabetic patients in a global context.

### 1.1 Objectives

1. To estimate the pooled prevalence of ED among diabetic patient in global context.
2. To identify the risk factors of ED among diabetic patient in global context.

## 2 METHODS

### 2.1 Protocol development and registration

This umbrella protocol was designed by preferred methods of reviewing available Systematic Review and Meta‐analysis (SRM) studies.^22^ First, the existence of a similar umbrella review was checked on PROSPERO, which found that there are no similar studies registered. Then, the protocol of this umbrella review was summited and registered (CRD42023488922) in PROSPERO. PROSPERO registration‐related information can be made available upon the reasonable request of the primary author. The umbrella review focuses on a systematic synthesis of existing systematic review and meta‐analysis studies toward the prevalence of ED and associated risk factors in diabetic patients worldwide.

### 2.2 Searching strategy and information sources

A compressive literature search will be conducted regarding systematic review and meta‐analysis of the prevalence of ED and associated risk factors in diabetic patients on Embase, Web of Sciences, PubMed, Cochrane Database of Systematic Reviews, Scopus, International Scientific Indexing (ISI), and Google Scholar using the PICO frameworks. Combining keywords and MeSH terms will be employed to retrieve the studies. Besides, the snowballing techniques will be utilized to retrieve additional studies in the citation list of articles found in the available database. Grey literature and manual search will also find unindexed/not published/research articles. Search strategies will be drafted using concepts and key search terms. The first concept will be: Erectile dysfunction: “erectile dysfunction,” “erectile problem,” “impotency,” “impotence,” “sexual impotence,” and “sexual dysfunction.” The second concept: Risk factors: “risk factors,” “associated factors,” “determinants,” “predictors,” and “cause.” The third concept: Diabetic: “diabetic,” “diabetes,” “diabetes mellitus,” and “DM.” The fourth concept: (SRM): “meta‐analysis,” ‘systematic review,” and “review.” Literature searching will be conducted by the two authors (T. A. K. and R. N. H.), independently. Any inconsistency will be resolved by agreement. In the case of article with incomplete information, the primary authors of the respective article will be contacted. We will use the search terms independently and/or in combination using “OR” or “AND”: (“erectile dysfunction” OR “erectile problem” OR “impotency” OR “impotence” OR “sexual impotence” OR “sexual dysfunction”) AND (“risk factors” OR “associated factors” OR “determinants” OR “predictors” OR “cause”) AND (“diabetic” OR “diabetes” OR “diabetes mellitus” OR “DM”) AND (“meta‐analysis” OR “systematic review” OR “review”). Besides, the “related article” and “cited by” features of the PubMed database will also be used to find articles from the included studies.

### 2.3 Eligibility criteria

#### 2.3.1 Inclusion criteria

A systemic review and meta‐analysis studies with reported prevalence and/or at least one associated risk factor of ED among diabetic patients written in the English language will be included. For the respective SRM to be considered for this umbrella review, it should fulfill the following prioritized criteria. (1) presented a defined literature search strategy, (2) appraised included studies using a relevant tool, and (3) followed a standard approach in pooling studies and providing summary estimates.

#### 2.3.2 Exclusion criteria

Articles will be excluded for one of the following reasons: (1) The article did not measure the outcome of interest for this umbrella review, (2) Article written other than in English language, (3) Narrative reviews, expert opinions, case reports, editorials, correspondence, abstracts, and methodological studies.

### 2.4 Data extraction and management

Two reviewers will conduct data extraction independently using a standardized extraction form. Screening and selection of articles will be first done through title and abstract, then after reviewing of full text will be done. In the cases of disagreement, discussion with other reviewers will be made to decide on the final article selection to include in this umbrella review. After the systemic search, the potentially eligible articles will be imported to EndNote 21. Duplicated studies will be removed in conditions where two or more articles have shared common characteristics. Structured data extraction in a Microsoft Excel spreadsheet will be prepared and used. The extracted data form includes (1) study identification (last name of the primary author and year of publications), (2) the aim and the type of review, (3) prevalence of ED, (4) odds ratio and 95%CI of risk factors of ED, (5) the number of included primary studies within SRM, (6) the number of sample size included, (7) publication bias and quality assessment technique, (8) analytic model type (fixed effect/random) and (9) the conclusion of the SRM.

### 2.5 Quality assessment (Risk of Bias Assessment)

The quality of the included article will be reviewed by two independent reviewers using the Assessment of Multiple Systematic Reviews (AMSTAR 2) tool. The new quality assessment tool extended from the previous AMSTAR. It contains 16 items to measure the whole approaches of the specific study review. The AMSTAR 2 tool is more robust and aggressive and minimizes quality scoring bias than the previous AMSTAR. The specific included article was extracted based on the 16 times of the AMSTAR 2 tool. The 16 article items will be filled into the online system on AMSTAR.com. The AMSTAR 2 automatically classify the level of evidence(quality) of specific SMR into four categories: (a) High‐quality evidence, (b) moderate‐quality evidence, (c) low‐quality evidence and (d) critically low‐quality evidence. Articles with critically low‐quality evidence will be excluded from this umbrella review.^23^

### 2.6 Quality of evidence assessment

We will use a powerful tool called GRADE tool (Grading of Recommendations Assessment, Development and Evaluation) to check how confident we can be in our results for each thing we are looking at. Assessments will be made for all five main domains (risk of bias, consistency, directness, precision, and publication bias), as well as the overall quality of evidence.^24^ In the end, we will give each outcome a score based on how strong the evidence is (Table 1).

**Table 1:** GRADE quality of evidence interpretations.

Grade	Definition
High	Further research is very unlikely to change our confidence in the estimate of effect.
Moderate	Further research is likely to have an important impact on our confidence in the estimate of effect and may change the estimate.
Low	Further research is very likely to have an important impact on our confidence in the estimate of effect and is likely to change the estimate.
Very low	Any estimate of effect is very uncertain

### 2.7 Dealing with duplicate and companion publications

We will prioritize a comprehensive data collection. In the event of duplicate publications, companion documents, or multiple reports of a primary study, we will utilize the most complete information available across all sources. Should any ambiguity remain, we may contact the author for clarification.

### 2.8 Statistical analysis

Once the data is extracted in Microsoft Excel, it will be imported to STATA version 17 software for analysis. Qualitative and narrative methods will be employed to present the summarized estimate of included studies. In a condition of two or more estimates on the same topic, the range of estimate and or pooled estimate will be used. Standard error will be computed by considering a binomial distribution formula. The overall prevalence of ED will be pooled using a random effect model.^25^ Besides, the pooled prevalence estimates and associated risk factors of ED will be presented by using a forest plot. In the case of risk factors, summary meta‐analytic estimates (i.e., odds ratios or relative risks) with 95% confidence intervals will be taken, and a pooled estimate will be generated using a random‐effects model. If the studies report relative risks but not odds ratios, they can be transformed into odds ratios. All the analysis and synthesis techniques will be similar to those used in pooled prevalence estimates. Cochrane's Q statistics (*χ* ^2^), inverse variance (*I* ^2^) and *p* values^26^ will be computed to show the level of heterogeneity between studies. Zero inverse variance (*I* ^2^) will reveal a true homogeneity, whereas 25%, 50%, and 75% show a low, moderate and high heterogeneity, respectively.^27^, ^28^ Subgroup analysis will be done using publication year, sample size, study quality (AMSTAR 2), IIEF‐6 (International Index of Erectile Function), IIEF‐15, single question, setting, and number of included studies. ED is also defined as severe (5–7), moderate (8–11), mild to moderate (12–16), mild (17–21), and no ED (22–25).^29^ Leave one out (sensitivity) meta‐analysis will be done to see the effect of a single study on overall pooled estimation. The funnel plot and Egger's regression test will be computed to identify the publication bias.^30^

### 2.9 Panel of experts

A carefully selected panel of experts in umbrella review methodology, statistics, and the relevant clinical field will be invited to engage in a structured discussion to reach a consensus on critical choices regarding the included systemic review and AMSTAR 2 quality assessment. Before the meeting, the panel will receive the current umbrella review protocol and any relevant supporting materials, such as preliminary findings and data summaries. The discussion will be facilitated to ensure diverse perspectives are heard and considered. Using a pre‐defined decision‐making process, the panel will address critical issues like inclusion/exclusion criteria for studies, interpretation of specific AMSTAR 2 criteria, and potential conflicts of interest. Their expertise and insights will be crucial in ensuring the highest quality and validity of the final umbrella review. The entire process will be transparently documented and communicated to stakeholders, including the rationale for decisions and supporting documentation.

## 3 DISCUSSION

This groundbreaking umbrella review promises to illuminate a hidden epidemic impacting millions of men globally: ED in diabetic populations. By meticulously reviewing and amalgamating existing evidence, we will be the first to comprehensively explore and map this critical issue globally. Our research delves into two crucial aspects: Unmasking the Global Burden: This review will serve as a powerful microscope, quantifying the true extent of ED among diabetic men across diverse regions and demographics. No longer will this issue remain shrouded in silence; we will shine a light on its staggering prevalence, urging the world to acknowledge its magnitude. Identifying the Culprits: Beyond simply mapping the problem, we will delve deeper and systematically analyze the risk factors associated with ED in diabetic patients. This could include age, duration of diabetes, specific health complications, lifestyle habits, and even psychosocial factors. Unveiling these culprits is crucial for developing targeted interventions and prevention strategies.

This umbrella review will build upon a robust systematic review and meta‐analysis encompassing global research on ED. It will offer a comprehensive understanding of the burden of ED and its associated risk factors in the global context. The research methodology will adhere to the rigorous PRISMA guidelines, guaranteeing the inclusion of high‐quality and relevant studies. Similarly, other umbrella review protocols^31^, ^32^, ^33^ also adhere with PRISMA guidelines. This might be due to the fact that, the PRISMA guidelines offer a standardized framework for reporting systematic reviews and meta‐analyses, enhancing transparency and credibility in research. By promoting thoroughness in documenting key aspects of the review process, PRISMA guidelines improve the quality of systematic reviews and facilitate interpretation of findings. Adherence to these guidelines reduces bias, enhances reproducibility, and facilitates efficient peer review. Ultimately, PRISMA guidelines contribute to evidence‐based practice by ensuring that research findings are based on rigorous methodology and transparent reporting.

This review will employ the innovative AMSTAR 2 tool to rigorously assess the methodological quality of each included study, providing an additional layer of confidence in the findings. However, most previous protocols did not implement AMSTAR 2.^34^, ^35^, ^36^ This variation in protocol adoption may stem from the fact that AMSTAR 2 is a relatively recent development and a revised instrument compared with its predecessor. As a result, some earlier protocols may not have incorporated AMSTAR 2 due to its emergence after their inception. In contrary recent protocols adopted AMSTAR 2 critical appraisal tool.^33^, ^37^, ^38^ This could be due to the fact AMSTAR 2 presents several strengths compared with its predecessor, AMSTAR 1. First, it reflects advancements in evidence synthesis methodology, incorporating updated methodological standards and criteria. This ensures a more rigorous evaluation of systematic review quality. Second, AMSTAR 2 provides clearer and more specific guidance on each assessment item, reducing ambiguity and enhancing consistency in appraisal among reviewers. Its nuanced rating system allows for finer distinctions between different levels of methodological rigor, unlike the binary scoring.

Although the search technique covers many terms related to ED and risk factors, reviews indexed using alternative terms may not be included. Moreover, umbrella reviews rely on information from review papers and may be limited by the quality of retrieved data. This limitation is also anticipated in other protocol.^36^ The study will only include articles written in the English language. Similarly, other protocols also plan to include articles only written in English.^31^, ^39^ Including only English articles in a review offers access to a vast array of high‐quality, peer‐reviewed literature, given English's dominance in scientific communication, notably in fields like medicine, psychology, and economics. However, this approach may limit inclusivity by excluding research conducted in other languages, potentially impacting representativeness.

The other anticipated limitations might be low‐medium quality level of some studies. This limitation is also consider in other protocol.^37^ Since we are planning to use AMSTAR 2 for critical appraisal of study quality, it is important to note that this tool tends to favor randomized controlled trials (RCTs) over observational studies. As a result, a review including only RCTs may receive a higher quality score, while a review incorporating primarily observational studies may receive a lower score. This disparity in scoring based on study design could potentially diminish the overall quality of evidence generated from the review. It highlights the need for careful consideration of study design diversity and the inclusion of various types of evidence to ensure a comprehensive and balanced assessment of the research topic.

Furthermore, this umbrella review also suffers from a common limitation observed in most umbrella reviews,^40^, ^41^, ^42^ wherein new and recent individual articles may not be included if they have not been incorporated into published systematic reviews and meta‐analyses. This restriction can result in the omission of potentially relevant studies that have emerged since the publication of the included reviews, thus limiting the comprehensiveness and currency of the synthesized evidence.

### 3.1 Review status

The project team planned to search relevant studies in the relevant databases from February 1 to February 29, 2024.

### 3.2 Protocol amendments

To ensure transparency and maintain scientific integrity, any significant changes to the protocol after study initiation requiring adjustments to eligibility, objectives, design, procedures, or analysis will be documented and require the consensus of all collaborators. This documentation will be readily available in a note attached to a future publication or a dedicated section within the study report detailing the discrepancies between the initial protocol and the final review.

## AUTHOR CONTRIBUTIONS

**Tegene Atamenta Kitaw**: Writing—original draft; conceptualization; validation; resources; investigation; methodology. **Biruk Beletew Abate**: Writing—review and editing; supervision; writing—original draft. **Befkad Derese Tilahun**: Methodology; validation; writing—review and editing; supervision. **Gizachew Yilak**: Conceptualization; methodology; validation. **Ribka Nigatu Haile**: Writing—review and editing; methodology; conceptualization; data curation; supervision.

## CONFLICT OF INTEREST STATEMENT

The authors declare no conflict of interest.

## TRANSPARENCY STATEMENT

The lead author Tegene Atamenta Kitaw affirms that this manuscript is an honest, accurate, and transparent account of the study being reported; that no important aspects of the study have been omitted; and that any discrepancies from the study as planned (and, if relevant, registered) have been explained.

## Supporting information

Supporting information.

## Data Availability

Not applicable.
