# Supplementary material for: Umbrella review protocol: Global burden and risk factors of erectile dysfunction in diabetic population
Source: Health Sci Rep. 2024 May 30;7(6):e2159. doi: 10.1002/hsr2.2159 (PMC11139671; doi:10.1002/hsr2.2159)
Supplement: Supplementary file 1 — Supporting information. [file HSR2-7-e2159-s001.doc]

**PRISMA-P (Preferred Reporting Items for Systematic review and Meta-Analysis Protocols) 2015 checklist: recommended items to address in a systematic review protocol***

| Section and topic | Item No | Checklist item |
| --- | --- | --- |
| ADMINISTRATIVE INFORMATION | | |
| Title: |  |  |
| Identification | 1 | An umbrella review protocol on estimating the global burden of erectile dysfunction and its associated risk factors in diabetic population. |
| Update |  |  |
| Registration | 2 | The protocol of this umbrella review was summited and registered (CRD42023488922) in PROSPERO. |
| Authors: |  |  |
| Contact | 3a | Tegene Atamenta kitaw: tegene2013@gmail.com  Biruk Beletew Abate: [birukkelemb@gmail.com](mailto:birukkelemb@gmail.com)  Befkad Derese Tilahun: [befkadderese6@gmail.com](mailto:befkadderese6@gmail.com)  Gizachew Yilak: [gyilak9@gmail.com](mailto:gyilak9@gmail.com)  Ribka Nigatu Haile: [nigaturebecca@gmail.com](mailto:nigaturebecca@gmail.com) |
| Contributions | 3b | All Authors- Tegene Atamenta kitaw, Biruk Beletew Abate, Befkad Derese Tilahun, Gizachew Yilak, Ribka Nigatu Haile contributed in conceptualization, methodology, writing and editing the protocol. |
| Amendments | 4 | To ensure transparency and maintain scientific integrity, any significant changes to the protocol after study initiation requiring adjustments to eligibility, objectives, design, procedures, or analysis will be documented and require the consensus of all collaborators. This documentation will be readily available in a note attached to a future publication or a dedicated section within the study report detailing the discrepancies between the initial protocol and the final review |
| Support: |  |  |
| Sources | 5a | There was no funding source for this study. |
| Sponsor | 5b | Not applicable. |
| Role of sponsor or funder | 5c | Not applicable. |
| INTRODUCTION | | |
| Rationale | 6 | Erectile dysfunction (ED) is a common complication of diabetes, affecting millions of men worldwide. Yet, despite its significant impact on diabetic patients, it remains largely a hidden burden, shrouded in stigma and shame. This lack of awareness and open discussion has far-reaching consequences, hindering early detection, treatment, and, ultimately, the prevention of long-term complications. Thus, understanding erectile dysfunction among diabetic patients in a global context can have contribute to improving the health and well-being of diabetic patients worldwide. It is also essential to gain global attention, promoting global collaboration to prevent the silent burden |
| Objectives | 7 | To estimate the pooled prevalence of erectile dysfunction among diabetic patient in global context.  To identify the risk factors of erectile dysfunction among diabetic patient in global context. |
| METHODS | | |
| Eligibility criteria | 8 | Inclusion criteria  A systemic review and meta-analysis studies with reported prevalence and/or at least one associated risk factor of erectile dysfunction among diabetic patients written in the English language will be included. For the respective SRM to be considered for this umbrella review, it should fulfil the following prioritized criteria. (1) presented a defined literature search strategy, (2) appraised included studies using a relevant tool, and (3) followed a standard approach in pooling studies and providing summary estimates.  Exclusion criteria  Articles will be excluded for one of the following reasons: (1) The article did not measure the outcome of interest for this umbrella review, (2) Article written other than in English language, (3) Narrative reviews, expert opinions, case reports, editorials, correspondence, abstracts, and methodological studies. |
| Information sources | 9 | A compressive literature search will be conducted regarding systematic review and meta-analysis of the prevalence of erectile dysfunction and associated risk factors in diabetic patients on Embase, Web of Sciences, PubMed, Cochrane Database of Systematic Reviews, Scopus, International Scientific Indexing (ISI), and Google Scholar |
| Search strategy | 10 | We will use the search terms independently and/or in combination using “OR” or “AND”: (‘erectile dysfunction’ OR ‘erectile problem’ OR ‘impotency’ OR ‘impotence’ OR ‘sexual impotence’ OR ‘sexual dysfunction’) AND (‘risk factors’ OR ‘associated factors’ OR ‘determinants’ OR ‘predictors OR ‘cause’) AND (‘diabetic’ OR ‘diabetes’ OR ‘diabetes mellitus’ OR ‘DM’) AND (‘meta-analysis’ OR ‘systematic review’ OR ‘review’). |
| Study records: |  |  |
| Data management | 11a | Screening and selection of articles will be first done through title and abstract, then after reviewing of full text will be done. In the cases of disagreement, discussion with other reviewers will be made to decide on the final article selection to include in this umbrella review |
| Selection process | 11b | Two reviewers will conduct data extraction independently using a standardized extraction form. After the systemic search, the potentially eligible articles will be imported to EndNote 21. Duplicated studies will be removed in conditions where two or more articles have shared common characteristics. Structured data extraction in a Microsoft Excel spreadsheet will be prepared and used |
| Data collection process | 11c | Two reviewers will conduct data extraction independently. |
| Data items | 12 | The extracted data form includes (1) study identification (last name of the primary author and year of publications), (2) the aim and the type of review, (3) prevalence of erectile dysfunction, (4) odds ratio and 95%CI of risk factors of erectile dysfunction, (5) the number of included primary studies within SRM, (6) the number of sample size included, (7) publication bias and quality assessment technique, (8) analytic model type (fixed effect/ random) and (9) the conclusion of the SRM. |
| Outcomes and prioritization | 13 | The two most prioritized outcome will be: (1) prevalence of erectile dysfunction in diabetic patient ,(2) risk factors of erectile dysfunction in diabetic patient. |
| Risk of bias in individual studies | 14 | The quality of the included article will be reviewed by two independent reviewers using the Assessment of Multiple Systematic Reviews (AMSTAR 2) tool. The new quality assessment tool extended from the previous AMSTAR. The AMSTAR 2 tool is more robust and aggressive and minimizes quality scoring bias than the previous AMSTAR. The specific included article was extracted based on the 16 times of the AMSTAR 2 tool. The 16 article items will be filled into the online system on AMSTAR.com. The AMSTAR 2 automatically classify the level of evidence(quality) of specific SMR into four categories: (a) High-quality evidence, (b) moderate-quality evidence, (c) low-quality evidence and (d) critically low-quality evidence. Articles with critically low-quality evidence will be excluded from this umbrella review (19). Furth more, subgroup analysis will be done based on the quality of included systemic review and meta-analysis |
| Data synthesis | 15a | Qualitative and narrative methods will be employed to present the summarized estimate of included studies. In a condition of two or more estimates on the same topic, the range of estimate and or pooled estimate will be used. Standard error will be computed by considering a binomial distribution formula. |
|  | 15b | The overall prevalence of erectile dysfunction will be pooled using a random effect model (20). Besides, the pooled prevalence estimates and associated risk factors of erectile dysfunction will be presented by using a forest plot. Cochrane’s Q statistics (Chi-square), inverse variance (I^2^) and p-values (21) will be computed to show the level of heterogeneity between studies. Zero inverse variance (I^2^) will reveal a true homogeneity, whereas 25%, 50%, and 75% show a low, moderate and high heterogeneity, respectively (22, 23) |
|  | 15c | Subgroup analysis will be done using publication year, sample size, study quality (AMSTAR 2), setting, and number of included studies. Leave one out (sensitivity) meta-analysis will be done to see the effect of a single study on overall pooled estimation. |
|  | 15d |  |
| Meta-bias(es) | 16 | The funnel plot and Egger’s regression test will be computed to identify the publication bias |
| Confidence in cumulative evidence | 17 | **We'll use a powerful tool called GRADE tool (Grading of Recommendations Assessment, Development and Evaluation) to check how confident we can be in our results for each thing we're looking at.** **Assessments will be made for all five main domains (risk of bias, consistency, directness, precision, and publication bias), as well as the overall quality of evidence.** In the end, we'll give each outcome a score based on how strong the evidence is. |

*** It is strongly recommended that this checklist be read in conjunction with the PRISMA-P Explanation and Elaboration (cite when available) for important clarification on the items. Amendments to a review protocol should be tracked and dated. The copyright for PRISMA-P (including checklist) is held by the PRISMA-P Group and is distributed under a Creative Commons Attribution Licence 4.0.**

*From: Shamseer L, Moher D, Clarke M, Ghersi D, Liberati A, Petticrew M, Shekelle P, Stewart L, PRISMA-P Group. Preferred reporting items for systematic review and meta-analysis protocols (PRISMA-P) 2015: elaboration and explanation. BMJ. 2015 Jan 2;349(jan02 1):g7647.*
